# Abducens Nerve Palsy as Initial Presentation of Multiple Myeloma and Intracranial Plasmacytoma

**DOI:** 10.3390/jcm7090253

**Published:** 2018-09-03

**Authors:** Elochukwu Ibekwe, Neil B. Horsley, Lan Jiang, Nadine-Stella Achenjang, Azubuogu Anudu, Zeeshan Akhtar, Karina G. Chornenka, Gregory P. Monohan, Yevgen G. Chornenkyy

**Affiliations:** 1 College of Medicine, University of Kentucky, Lexington, KY 40506, USA; ibekwe@uky.edu (E.I.); neil.horsley@uky.edu (N.B.H.); lan.jiang@uky.edu (L.J.); stella.achenjang@uky.edu (N.-S.A.); azu.anudu@uky.edu (A.A.); zeeshan.akhtar@uky.edu (Z.A.); 2 Faculty of Medicine, University of British Columbia, Vancouver, BC V6T 1Z4, Canada; k.chornenka@alumni.ubc.ca; 3 Department of Hematology-Oncology, University of Kentucky Medical Center, Lexington, KY 40536, USA; gpmono0@email.uky.edu; 4 Department of Molecular and Cellular Biochemistry, University of Kentucky, Lexington, KY 40536, USA

**Keywords:** multiple myeloma, plasmacytoma, abducens nerve palsy

## Abstract

Central Nervous System (CNS) involvement in multiple myeloma and/or multifocal solitary plasmacytoma is rare. Although they are unique entities, multiple myeloma (MM) and plasmacytoma represent a spectrum of plasma cell neoplastic diseases that can sometimes occur concurrently. Plasmacytomas very often present as late-stage sequelae of MM. In this case report, we report a 53-year-old female presenting with right abducens cranial nerve (CN) VI palsy as an initial presentation secondary to lesion of the right clivus.

## 1. Introduction

Central Nervous System (CNS) involvement in multiple myeloma (MM) is uncommon, with an incidence of approximately 1% of all MM cases [1,2]. CNS involvement suggests the presence of either advanced stage sequela of MM known as intracranial plasmacytomas (occurring in up to 5% of patients) [3], or solitary plasmacytoma without evidence of systemic disease. Intracranial plasmacytomas are more common in the base of the skull and theyoriginate from bone or paranasal sinus soft tissue. Initial symptoms can manifest as isolated or multiple cranial nerve (CN) deficits. 

MM and intracranial plasmacytoma represent a spectrum of plasma cell neoplasm. MM typically presents with bone pain secondary to lytic bone lesions, an increase in serum and/or urine monoclonal protein concentration, hypercalcemia, anemia, persistent or recurrent infections, and/or acute renal failure [4]. In contrast, solitary plasmacytomas are localized plasma cell tumors arising from soft tissue (extramedullary plasmacytomas) or bones (solitary bone plasmacytomas) without evidence of systemic MM disease [5,6]. While CNS involvement in MM is rare, it is sometimes the only clinical finding initially present. It is important to recognize that plasmacytoma can be associated with MM and early recognition will hasten intervention. Presented is a case of intracranial plasmacytoma associated with MM in a patient who presented with abducens nerve (CN VI) palsy as the initial clinical manifestation of the disease.

## 2. Case Presentation 

A 53-year-old female with a history of type 2 diabetes mellitus, hypertension, and hypothyroidism presented with increasing diplopia and nausea for six days. The patient experienced an unintentional weight loss of 10 pounds in the preceding two weeks. Physical exam revealed bony tenderness localized to the ribs as well as a right CN VI palsy manifesting as impaired right eye abduction. Remainder of the exam was unremarkable.

Hemogram with differential was remarkable for white count of 10,700 k/µL (3.7–10.3 k/µL), with absolute neutrophil count of 7.5 k/µL (1.6–6.1 k/µL). Absolute lymphocyte count was within normal limits (2.42 k/µL (1.6–6.1 k/µL)). Blood chemistry was remarkable for: serum Ca^2+^ of 15.8 mg/dL (8.9–10.2 mg/dL), ionized Ca^2+^ of 7.7 mg/dL (4.6–5.1 mg/dL), and glucose of 254 mg/dL (90–120 mg/dL). Parathyroid hormone was <10 pg/mL (12–72 pg/mL) and parathyroid hormone related peptide was 1.3 (normal). Urine analysis and urine protein electrophoresis was unremarkable without evidence of Bence-Jones protein. The calculated protein gap between total protein (6.8 g/dL) and serum globulin (2.8 g/dL) was 4.0 g/dL and the albumin/gamma globulin ratio was elevated at 2.4 (0.8–2.0). 

Serum protein electrophoresis revealed faint monoclonal immunoglobulin. Serum immune-quantification showed IgG 1150 mg/dL (720–1598 mg/dL), IgA 200 mg/dL (75–400 mg/dL), and IgM 41 (35–225 mg/dL). Kappa light chain was 108.18 mg/L (3.30–19.4 mg/L), lambda light chain 445.32 mg/L (5.71–26.30 mg/L), with a kappa/lambda free light chain ratio of 0.24 (0.26–1.65). Bone marrow biopsy results demonstrated hypercellular bone marrow involved by plasma cell neoplasm (50–60% aberrant lambda restricted plasma cells). Fluorescence in situ hybridization studies found evidence of CCND1/IGH gene fusion and gain of chromosome 1q. Flow cytometry of bone marrow aspirate demonstrated a small population of aberrant lambda restricted plasma cells positive for CD38, CD56, moderate CD45 and Lambda, and negative for CD19.

**Figure 1:**
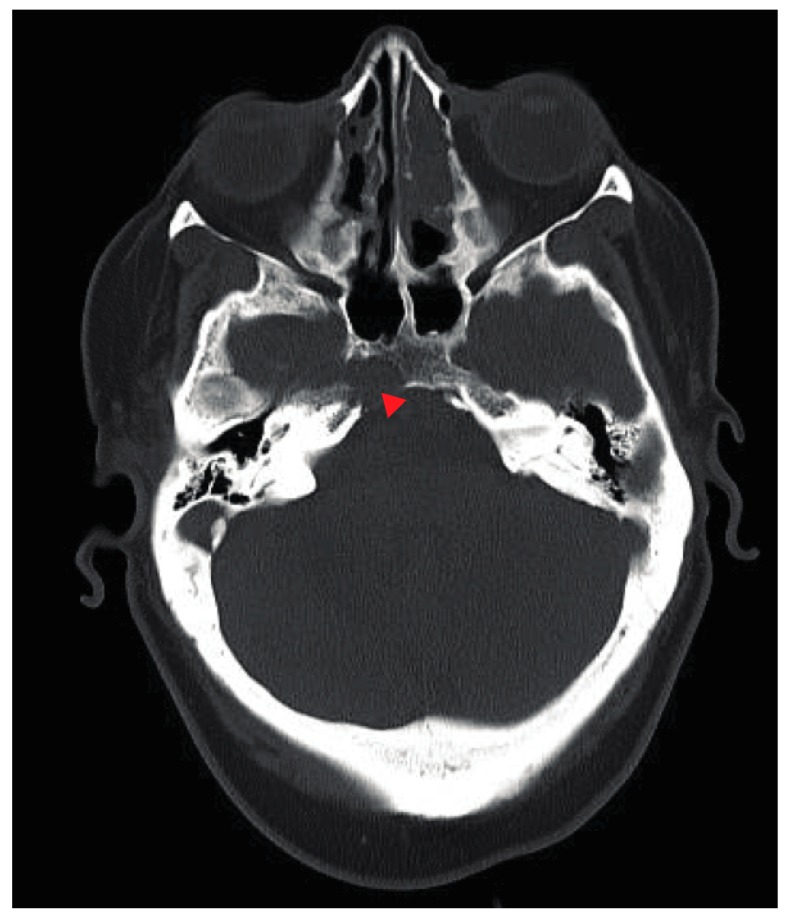
Axial CT head demonstrating lytic bone lesion (indicated by red triangle) within the right side of the clivus adjacent to Dorello’s canal likely accounting for the patient’s 6th nerve palsy.

**Figure 2:**
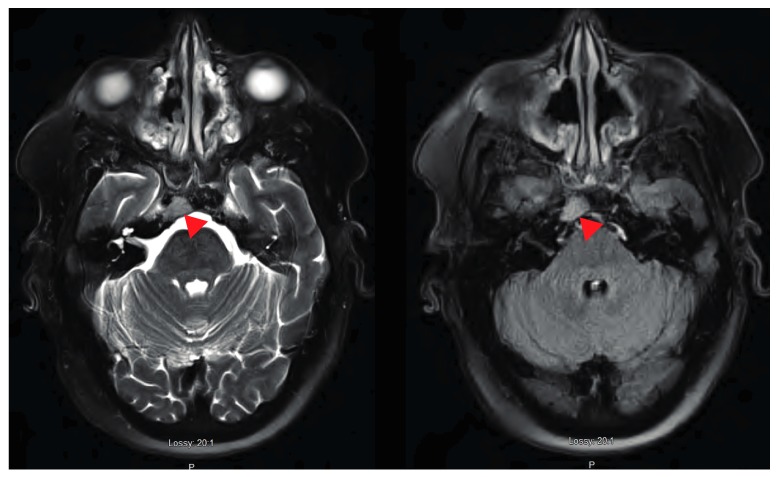
Axial T2 FS repeat (**left**) and T2 (**right**) demonstrating a discrete lesion (indicated by red triangle) in the same region as CT scan. Lesion is seen on the right clivus adjascent to Dorello’s canal and is likely responsible for CN 6 palsy. Lesion suspicious for myeloma and metastases. However, given the signal intensity and restricted diffusion lymphoma is also a possibility.

**Figure 3:**
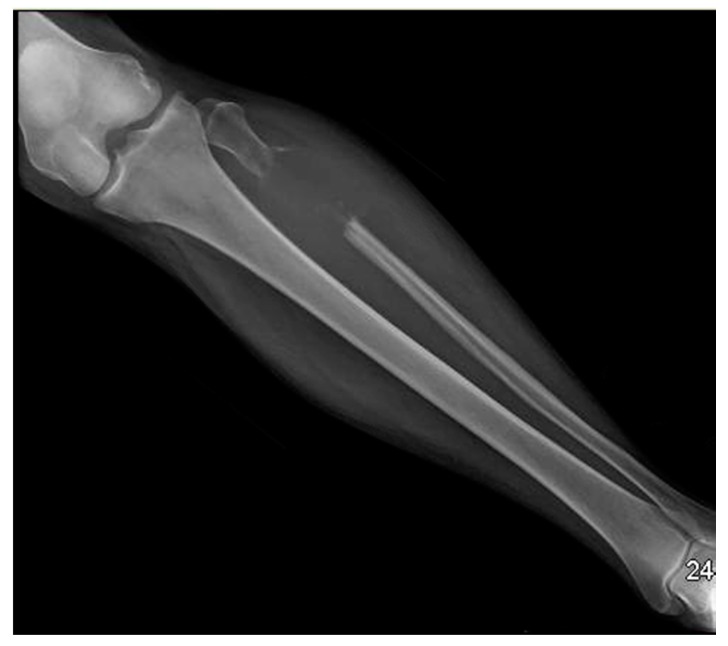
Bone survey demonstrated lytic lesions in the left proximal fibular diaphysis.

Computed tomography (CT) of the chest/abdomen revealed multiple osteolytic lesions in the appendicular and axillary skeleton throughout the thoracolumbar vertebral bodies, and pelvic bones; a healing non-displaced fifth anterior lateral rib fracture was present on the right. CT and magnetic resonance imaging (MRI) of the head showed multiple bone lesions, with a well-defined lesion measuring 12 × 15 mm within the right side of the clivus adjacent to Dorello’s canal (Figure 1 and Figure 2). Bone survey showed lytic lesions in the left proximal fibular diaphysis (Figure 3). 

The patient’s hypercalcemia was treated with IV normal saline, calcitonin, and pamidronate. Pain was controlled with acetaminophen and tramadol with oxycodone for breakthrough pain. The patient was evaluated by the hematology-oncology service and received external beam radiation therapy to the clivus, alleviating her CN VI palsy. Her disease was then managed with cyclophosphamide, bortezomib, and dexamethasone (CyBorD) chemotherapy. With treatment her symptoms resolved. Three months into therapy, her repeat laboratory testing demonstrated good response to treatment. The lambda light chain had decreased to 13.1 mg/L (5.71–26.30 mg/L), with a normal kappa/lambda free light chain ratio of 1.0 (0.26–1.65). Furthermore, a repeat bone marrow biopsy was obtained, which showed no morphologic evidence of multiple myeloma.

## 3. Discussion

MM and plasmacytoma are a spectrum of plasma cell neoplastic disease, with MM accounting for about one percent of all cancers in the USA and is the second most common hematologic malignancy after lymphoma. In 2016, there were an estimated 30,300 new cases, 12,650 deaths, and an incidence of about 4–5 per 100,000 in the United States (US) [7]. Plasmacytoma constitutes less than 1% of intracranial tumors and can occur intramedullary or extramedullary. It can occur as a primary or secondary malignancy to widespread MM and can be classified into three groups. (1) plasmacytoma arising from the skull base; (2) plasmacytoma involving brain parenchyma +/− skull involvement termed “intracranial tumor syndromes”; and, (3) plasmacytomas involving the orbit termed “intraorbital tumor syndromes” [8]. Although plasmacytoma is rare, early recognition is vital in advanced MM disease as it is associated with increased morbidity and mortality when diagnosis is delayed. 

MM is believed to result from malignant transformation of post-germinal center plasma cells producing a monoclonal immunoglobulin, while plasmacytoma are tumors of plasma cells identical to that seen in MM [9]. Plasmacytomas that occur in the brain are termed intracranial plasmacytoma, and can arise from: (1) the clivus bone, which forms the junction between the basilar occipital and the sphenoid bone, (2) petrous part of the temporal bone, (3) or an extension of a plasmacytoma tumor arising from the submucosa of the nasopharyngeal region [3,8,10]. Due to the rarity of plasmacytomas, it is important to consider other differential diagnoses that include chondrosarcomas and meningiomas, and osteosarcomas and Ewing’s tumor for a plasmacytoma arising from the clivus and petrous bone, respectively [3,11]. 

The symptoms of a plasmacytoma depend on the location and size of the tumor. Patients may present with headache, seizures, focal neurologic deficits, or cranial nerve palsies [11]. Although any cranial nerve can be affected, unilateral CN VI palsy is a common presentation [8]. This is due to the extended course taken by CN VI between the pons and the clivus to pierce the dura mater between the dura and skull. To achieve this, CN VI fibers pass via Dorello’s canal and bend at the petrous temporal bone, traveling into the superior orbital fissure to innervate the lateral rectus muscle of the eye. Patients with CN VI deficits will present with diplopia due to the inability to abduct the affected eye. Other nerves commonly affected include CN II and III [12]. 

As previously mentioned, a plasmacytoma can be solitary or secondary to MM. Diagnosis of a solitary plasmacytoma depends on positive biopsy-proven clonal plasma cells solitary lesion without any evidence of systemic MM disease. Diagnosis is confirmed with lytic homogenous, soft-tissue density on CT or MRI. MRI is favored over CT for tumor imaging and it will show isointense focus [13]. Plasmacytomas can also arise in the context of MM [14].

Molecular characterization of MM can provide useful clinical information regarding prognosis and therapy stratification. The presence of specific cytogenetic high-risk abnormalities, including t(4;14), t(14;16), t(14;20), del(17p), and dup(1q) were previously identified to confer a poor prognosis [15,16,17]. On cytogenetic analysis, our patient was found to have CCND1/IGH gene fusion, which is commonly known as t(11;14) fusion and dup(1q). It should be noted that t(11:14) is a neutral or favorable prognostic factor in MM; however, in patients with light chain amyloidosis it confers a hazard ratio for death of 2.5 [18]. Duplications of 1q are considered secondary genetic abnormalities in MM and are typically associated with disease progression, high-risk disease, and shorter survival [17]. This is likely secondary to the fact that duplications of 1q reflect an increase in the gene dosage of proliferative genes, like CKS1B [16,17,19].

Clinically, it is difficult to ascertain whether a plasmacytoma is a clinical sequela of MM or if the plasmacytoma progressed to MM. Nevertheless, both circumstances carry a worse prognosis and should be aggressively treated. Treatment consists of localized radiotherapy for plasmacytoma along with systemic chemotherapy for concurrent MM. The choice of chemotherapy regimen depends on MM risk stratification; combination chemotherapy regimen, such as bortezomib, lenalidomide, and low dose dexamethasone are often recommended initial agents for treatment [20]. 

## 4. Conclusions

CNS involvement in MM is rare. It commonly manifests as an intracranial plasmacytoma and it typically indicates late stage MM with poor prognosis. The initial clinical presentation includes diplopia, bone pain, and weight loss. Although a solitary plasmacytoma can manifest without MM co-morbidity, it can progress to MM [11]. Thus, prompt recognition and treatment of the plasmacytoma is warranted. 

Our patient’s chief complaint was diplopia, with clinical findings of hypercalcemia and a clivus bone lytic lesion being discovered on workup. Therefore, it is important to consider conducting an MM workup in any patient presenting with diplopia and an accompanying lytic bone lesion found on imaging.
